# LRP6/filamentous-actin signaling facilitates osteogenic commitment in mechanically induced periodontal ligament stem cells

**DOI:** 10.1186/s11658-023-00420-5

**Published:** 2023-01-24

**Authors:** Jixiao Wang, Huiqi Yang, Xiaobei Ma, Jiani Liu, Lan Li, Lei Chen, Fulan Wei

**Affiliations:** Department of Orthodontics, School and Hospital of Stomatology, Cheeloo College of Medicine, Shandong University & Shandong Key Laboratory of Oral Tissue Regeneration & Shandong Engineering Laboratory for Dental Materials and Oral Tissue Regeneration & Shandong Provincial Clinical Research Center for Oral Diseases, No. 44-1 Wenhua Road West, Jinan, 250012 Shandong China

**Keywords:** Mechanical stress, PDLSCs, Mechanotransduction, LRP6, Filamentous actin, Osteogenic commitment

## Abstract

**Background:**

Mechanotransduction mechanisms whereby periodontal ligament stem cells (PDLSCs) translate mechanical stress into biochemical signals and thereby trigger osteogenic programs necessary for alveolar bone remodeling are being deciphered. Low-density lipoprotein receptor-related protein 6 (LRP6), a Wnt transmembrane receptor, has been qualified as a key monitor for mechanical cues. However, the role of LRP6 in the mechanotransduction of mechanically induced PDLSCs remains obscure.

**Methods:**

The Tension System and tooth movement model were established to determine the expression profile of LRP6. The loss-of-function assay was used to investigate the role of LRP6 on force-regulated osteogenic commitment in PDLSCs. The ability of osteogenic differentiation and proliferation was estimated by alkaline phosphatase (ALP) staining, ALP activity assay, western blotting, quantitative real-time PCR (qRT-PCR), and immunofluorescence. Crystalline violet staining was used to visualize cell morphological change. Western blotting, qRT-PCR, and phalloidin staining were adopted to affirm filamentous actin (F-actin) alteration. YAP nucleoplasmic localization was assessed by immunofluorescence and western blotting. YAP transcriptional response was evaluated by qRT-PCR. Cytochalasin D was used to determine the effects of F-actin on osteogenic commitment and YAP switch behavior in mechanically induced PDLSCs.

**Results:**

LRP6 was robustly activated in mechanically induced PDLSCs and PDL tissues. LRP6 deficiency impeded force-dependent osteogenic differentiation and proliferation in PDLSCs. Intriguingly, LRP6 loss caused cell morphological aberration, F-actin dynamics disruption, YAP nucleoplasmic relocation, and subsequent YAP inactivation. Moreover, disrupted F-actin dynamics inhibited osteogenic differentiation, proliferation, YAP nuclear translocation, and YAP activation in mechanically induced PDLSCs.

**Conclusions:**

We identified that LRP6 in PDLSCs acted as the mechanosensor regulating mechanical stress-inducible osteogenic commitment via the F-actin/YAP cascade. Targeting LRP6 for controlling alveolar bone remodeling may be a prospective therapy to attenuate relapse of orthodontic treatment.

**Supplementary Information:**

The online version contains supplementary material available at 10.1186/s11658-023-00420-5.

## Background

Alveolar bone can experience a remodeling process in response to mechanical stress from orthodontic loads to establish new homeostasis, which is mainly influenced by mechano-adaptive events from the periodontium [1, 2]. PDLSCs, the main functional osteoprogenitors in the periodontal ligament (PDL), are involved in the adaptive activities by mechanosensing and signaling [3]. Specifically, PDLSCs perceive biophysical stimuli and convert them into the osteogenic niche, which directly acts on cellular self-renewal and differentiation and consequently on adjacent alveolar bone turnover [4, 5]. To date, extensive literature including our previous studies has been devoted to delineating how stressed PDLSCs sense, respond, and transduce the propagated signals [6–8]. Indeed, our insights into force-instigated intracellular signaling cascades that regulate osteogenic activities in PDLSCs have gained momentum [9]. However, knowledge on mechanosensors that link extrinsic stress to intrinsic biochemical response is limited. Identifying mechanosensors that initiate mechano-to-chemo signaling events would provide a broader horizon to map the full-scale mechanical landscape in PDLSCs.

Generally, cell surface receptors are regarded as the overarching mechanosensors in mechanosensory cells, as they are the first response to changes in the microenvironment [10, 11]. Although several membrane-embedded proteins for controlling osteogenic commitment in force-induced PDLSCs have been identified [12–14], many of the sensory elements on, or spanning, the cell membrane remain to be defined. LRP6 is a Wnt transmembrane receptor that plays a range of important functions in cell mechanosensing by activating the Wnt/β-Catenin signaling [15, 16]. Recently, LRP6 was discovered as a critical regulator participating in dental physiological and pathophysiological activities such as tooth development, oligodontia, and oral squamous cell carcinoma [17, 18]. Notably, the mutation of low-density lipoprotein receptor-related protein 5 (LRP5), which shares 71% identity of the amino acid sequences with LRP6, decreased the PDL width and destroyed the periodontal complex [19, 20]. As such, we speculate that LRP6 in PDLSCs is a candidate response sensor that answers to mechanical stress and mediates osteogenic commitment.

This study aimed to illuminate key sensors mechanosignaling that regulate osteogenic activities in mechanically induced PDLSCs during orthodontic tooth movement (OTM). We found that LRP6 reacted to mechanical stress and effectually mediated downstream osteogenic commitment via the F-actin/YAP cascade in PDLSCs. We thus proposed a novel mechanism for LRP6 beyond the Wnt/β-Catenin signaling to regulate osteogenic activities. Furthermore, exploiting mechanotherapeutics that specifically target LRP6 may provide a promising avenue for anchorage maintenance and relapse reduction during OTM.

## Methods

### Cell cultivation and identification

All experiments for processing human PDL tissues were ratified by the Ethics Committee of School and Hospital of Stomatology, Cheeloo College of Medicine, Shandong University. Informed consent was acquired from all participants as well as their guardians. Healthy third molars and premolars were extracted from disease-free participants aged 16–25 for orthodontic needs. The PDL was gently scraped from the middle part of the dental root surface. Human PDLSCs were derived and cultured by the explant culture method, as previously reported [21]. Cells from passages 3–5 were employed in the following studies. The surface markers of cells at passage 4 were determined and analyzed by flow cytometry (Becton Dickinson, Franklin Lakes, USA) according to the manufacturer’s instructions. Crystal violet staining (Solarbio, Beijing, China) was performed to evaluate the colony-forming ability of cells.

### Multidirectional differentiation of PDLSCs

Cells were cultured in the osteogenic-/adipogenic-inducing medium to confirm the capability of multidirectional differentiation. The components of the inducing medium were described in our previous research [21]. After induction on day 21, 4% paraformaldehyde was used to fix cells. Then, Alizarin Red Staining kit (Sigma-Aldrich, St. Louis, USA) and Oil Red O kit (Solarbio, Beijing, China) were implemented to stain cells following the instructions of the manufacturers.

### Cyclic stretch stress (CSS) application

CSS (10% elongation, 0.5 Hz) was performed using the Flexcell FX-5000 Tension System (Flexcell International Corp., Burlington, USA) to mimic appropriate orthodontic force in vivo.

**Fig. 1:**
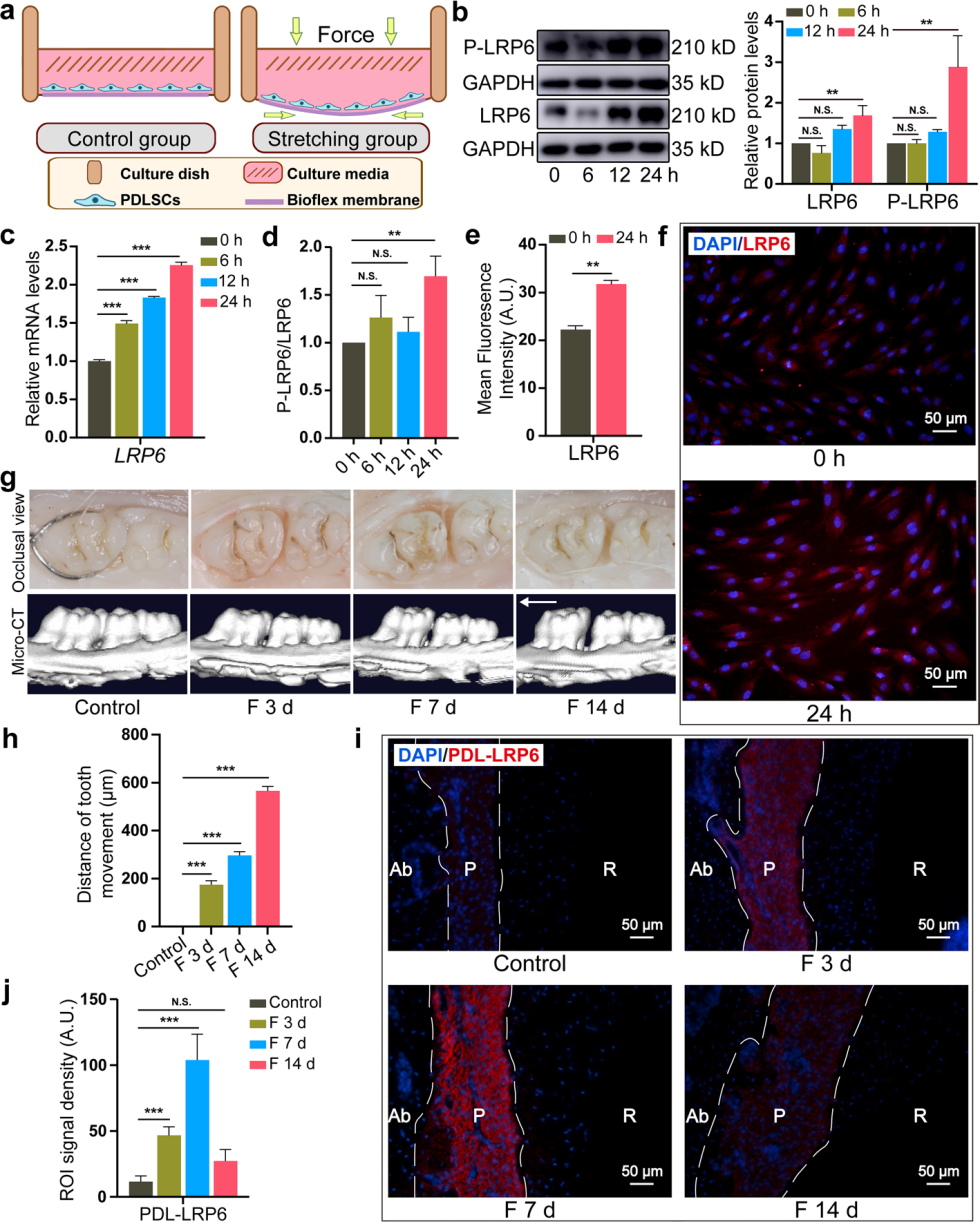
Enhanced LRP6 expression in force-induced PDLSCs and PDL tissues. **a** Diagram of the in vitro cell tension-loading system. **b** The protein expression of LRP6 and P-LRP6 in PDLSCs during CSS loading. **c** The mRNA expression of *LRP6* in PDLSCs during CSS loading. **d** The ratio of the protein levels of P-LRP6/total LRP6 in PDLSCs during CSS loading. **e**, **f** Immunofluorescence and semi-quantitative analysis of LRP6 expression under CSS for 24 h. Scale bar, 50 μm. **g** Representative occlusal view and micro-computed tomography of OTM for 3, 7, and 14 days (F 3 d, F 7 d, F 14 d, *N* = 5). The arrow represents the direction of force loading. **h** Quantitative analysis of the change in the distance of OTM. *N* = 5. **i**, **j** Immunofluorescence and semi-quantitative analysis of LRP6 expression in the periodontium during mechanical force application. *N* = 5. Ab, alveolar bone; P, periodontal tissue; R, root. Dotted lines mark the boundary of the PDL. Scale bar, 50 μm. *P < 0.05, **P < 0.01, ***P < 0.001. N.S., no significance. Results are presented as mean ± SD of triplicated experiments

A schematic cartoon is shown in Fig. 1a. A total of 20 × 10^4^ cells per well were seeded into the Bioflex culture plates (Flexcell International Corp., Burlington, USA) and incubated to 80% confluence. The serum-deprived medium (2% fetal bovine serum) was used to culture cells for 24 h before stretching. Cells were cultivated in the same parameters without stretching as the control group.

### Cell morphology analysis

The cells after stretching were washed with PBS and fixed with 4% paraformaldehyde. To stain the shape of PDLSCs, 0.1% Crystal Violet Stain Solution (Solarbio, Beijing, China) was used. The Leica DMi8 microsystems (Lecia, Wetzlar, Germany) were utilized to image cells. Five random fields were selected per well, and ten cells were randomly chosen in every field to measure and analyze. Cellular phenotyping data including cell area, roundness, and width-to-length ratio were determined and analyzed using FIJI (NIH, USA) by thresholding.

### Lentivirus transfection

Green fluorescent protein-labeled lentiviral construct encoding a short hairpin RNA-targeted LRP6 (named sh-LRP6) was designed and produced by Shanghai Zorinbio Co., Ltd. (Shanghai, China). The unique construct was reported in a previous study [22]. The identical lentiviral vector carrying an insert of nonspecific RNA oligonucleotide was used as the negative group named sh-NC. An appropriate multiplicity of infection (MOI, 10) was adopted to transfect lentivirus into cells with the help of polybrene (10 μg/ml). Cells without lentivirus transfection were divided into the blank control group. After transfection for 72 h, cells from three groups were exposed to CSS for 24 h.

### RNA extraction and quantitative real-time PCR (qRT-PCR)

Cells were washed twice with PBS after stretching. Total cellular RNA was collected utilizing RNAiso Plus (Takara Bio Inc., Shiga, Japan). cDNA was synthesized from total cellular RNA (10 μg) by PrimeScriptTM RT Master Mix (Takara Bio Inc., Shiga, Japan). Then Roche LightCycler 480 system (Roche, Mannheim, Germany) was adopted to perform qRT-PCR and analyze the mRNA expression. Triplicate data were analyzed using the 2^−ΔΔCT^ method, and GAPDH was adopted as the normalized reference. The primer sequences are presented in Additional file 1: Table S1.

### Western blotting

Cells were washed thrice with PBS after stretching. The lysis buffer with 1% PMSF and 1% phosphatase inhibitor was used to extract cellular protein on ice. Protein was separated by 8% SDS polyacrylamide gel electrophoresis and transferred onto 0.2 μm polyvinylidene difluoride membranes. Then, the membranes were incubated overnight at 4 °C with the primary antibodies after 5% (w/v) skimmed milk blocking for 1 h. HRP-conjugated secondary antibodies (1:8000, Proteintech, Wuhan, China) were subsequently incubated with the membranes at room temperature for 1 h. The immunoreactive bands were detected by ECL chromogenic substrate. GAPDH was adopted as an internal standard. The ImageJ software (NIH, USA) was used to quantize the densitometric data.

### ALP staining and ALP activity assay

After stretching for 24 h, cells were washed with PBS and fixed with 4% paraformaldehyde. ALP staining reagent (Beyotime, Shanghai, China) was incubated with cells for 15 min to visualize cellular ALP. The ALP activity was measured by ALP activity kit (Jiancheng bio-engineering institute, Nanjing, China) following the manufacturers’ instructions.

### Immunofluorescence staining

We used PBS to wash cells and then 4% paraformaldehyde to fix cells for 20 min after stretching. Triton X-100 (0.1%, Solarbio, Beijing, China) was used to permeate the cytomembrane. Cells were subsequently blocked with goat serum (10%, Solarbio, Beijing, China) for 1 h. Then, the primary antibodies were incubated with cells overnight at 4 °C. The secondary antibodies were incubated with cells for 1 h at room temperature after washing twice. DAPI was used to stain the cell nucleus. For 63× microscopy images, silicone membranes were cut from Bioflex culture plates and transferred to glass slides, and sealed with mounting medium (Abcam, Cambridge, UK). The Leica DMi8 microsystems (Lecia, Wetzlar, Germany) were utilized to visualize and picture cells. Semi-quantification analysis was implemented by the ImageJ software.

### Orthodontic tooth movement model built

The experiment was approved by the Ethics Committee of School and Hospital of Stomatology, Cheeloo College of Medicine, Shandong University. All operations abided by the National Institutes of Health Guide for the Care and Use of Laboratory Animals. The tooth movement model was constructed using 8-week-old male Wistar rats (Pengyue, Jinan, China). The nickel–titanium coiled springs (TOMY, Fukushima, Japan), which deliver a mesial force of 25 g, were ligated between the right maxillary molar and maxillary incisor (Additional file 2: Fig. S1). The maxillary first molar (M1) was mesially moved for 0, 3, 7, and 14 days using the maxillary incisor as the anchorage. The left dentition was considered as the control. Semi-quantification of positive cells (*N* = 5) was analyzed by the ImageJ software. Hematoxylin–eosin (HE) staining (Solarbio, Beijing, China) was used to observe the morphological change of the PDL on the tension side of M1 mesiobuccal roots during OTM.

### Chemicals and reagents

The primary antibodies are shown in Additional file 1: Table S2. The original images of western blotting of primary antibodies are presented in Additional file 3. Actin-Tracker-Red-555 (1:100, Beyotime, Shanghai, China) was used to stain cellular F-actin. Cytochalasin D (Cyto D, 0.2 μg/ml, MedChemExpress, NJ, USA) dissolved in DMSO was used to disrupt cellular F-actin.

### Statistical analysis

Experimental data with a normal distribution are shown as mean ± standard deviation(s) per group, which includes three or more independent samples by using the GraphPad Prism 9.0 software (GraphPad Software Inc., San Diego, USA). Significant variances between different groups are displayed by one-way ANOVA or Student’s *t*-test after Tukey’s post hoc analysis. Variances with *P* < 0.05 were regarded as statistically significant.

## Results

### Enhanced LRP6 expression in force-induced PDLSCs and PDL tissues

The expression profile of LRP6 was explored by the Tension System (Fig. 1a) and the tooth movement model. In vitro, we exposed cultured cells to CSS for 6, 12, and 24 h, simulating in vivo mechanical force loading. The expression of LRP6 exhibited time-dependent upregulation compared with the no-stretch group and stretching for 24 h manifested the most significant elevation (Fig. 1b, c). Phosphorylated LRP6 (P-LRP6) exhibited the same tendency (Fig. 1b). CSS application upregulated the ratio of the protein levels of P-LRP6/total LRP6, and the most significant change was observed at 24 h (Fig. 1d). Immunofluorescence confirmed that LRP6 was dramatically increased after stretching for 24 h (Fig. 1e, f). Stretching for 24 h was thus adopted in the succedent study. The data suggested that LRP6 may be responsible for the response of PDLSCs to mechanical force. Moreover, micro-CT (Fig. 1g) and the distance of tooth movement (Fig. 1h) indicated that the tooth movement model was appropriate. HE staining showed the in vivo distraction of the tension side of PDL tissues (Additional file 2: Fig. S2). We observed that LRP6 expression was distributed throughout the periodontium, and its expression was gradually upregulated from day 0 to day 7. On day 14, LRP6 expression descended compared with that on day 3 and day 7 (Fig. 1i, j). The data further hinted that LRP6 might participate in the alveolar bone remodeling during OTM. Besides, LRP5 and LRP6, which act as Wnt co-receptors, share 71% homology. We also found that LRP5 in PDLSCs was also upregulated under CSS (Additional file 2: Fig. S3).

### LRP6 inactivation inhibited osteogenic commitment in force-induced PDLSCs

**Fig. 2:**
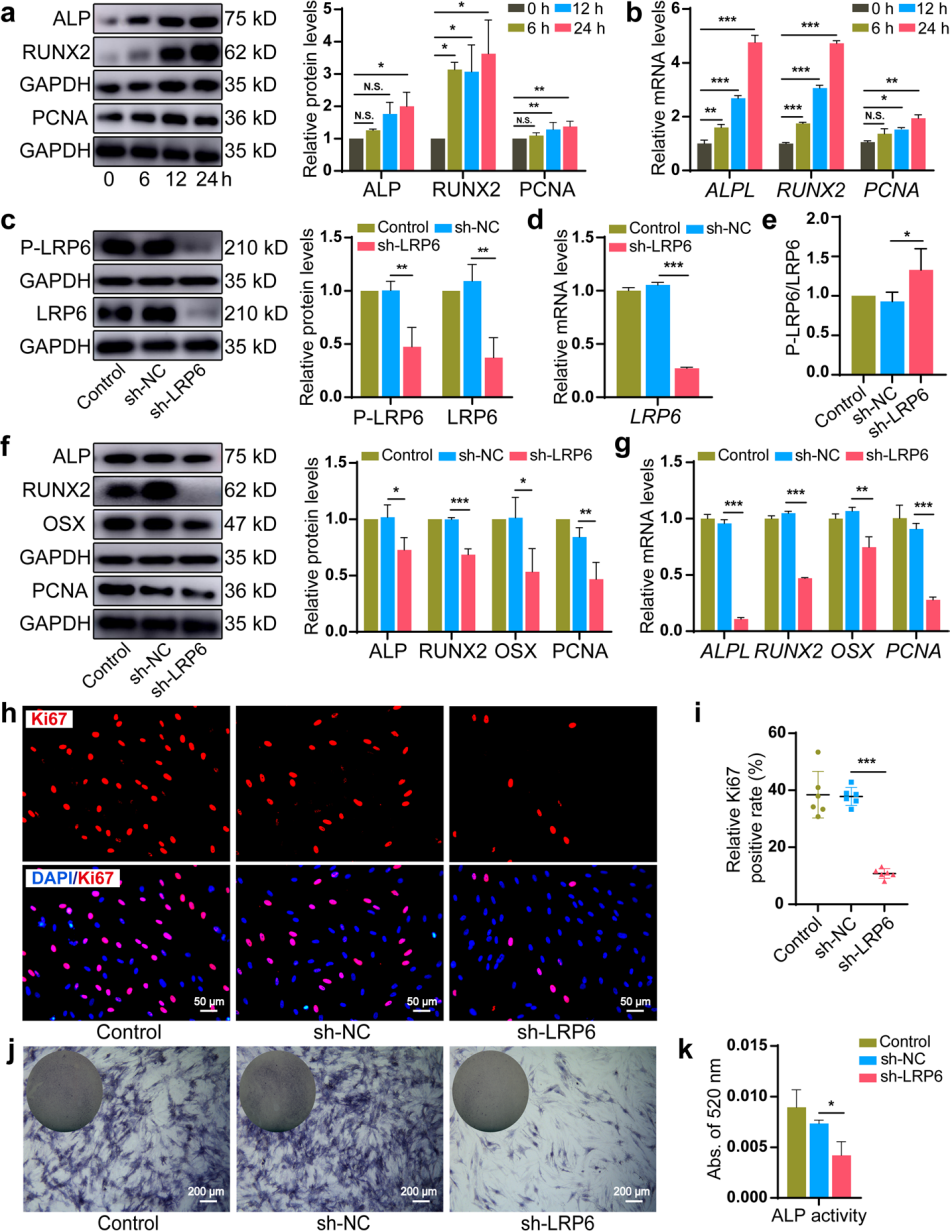
LRP6 inactivation inhibited osteogenic commitment in force-induced PDLSCs. **a** The protein expression of ALP, RUNX2, and PCNA in PDLSCs under CSS. **b** The mRNA expression of *ALPL*, *RUNX2*, and *PCNA* in PDLSCs under CSS. **c** The protein expression of P-LRP6 and LRP6 in the control, sh-NC, and sh-LRP6 group after stretching for 24 h. **d** The mRNA expression of *LRP6* in the control, sh-NC, and sh-LRP6 group after stretching for 24 h. **e** The ratio of the protein levels of P-LRP6/total LRP6 in the control, sh-NC, and sh-LRP6 group after stretching for 24 h. **f** The protein expression of ALP, RUNX2, OSX, and PCNA in the control, sh-NC, and sh-LRP6 group after stretching for 24 h. **g** The mRNA expression of *ALPL*, *RUNX2*, *OSX*, and *PCNA* in the control, sh-NC, and sh-LRP6 group after stretching for 24 h. **h**, **i** Immunofluorescence and semi-quantitative analysis of Ki67^+^ cells in the control, sh-NC, and sh-LRP6 group after stretching for 24 h. Scale bar, 50 μm. **j**, **k** ALP staining and ALP activity analysis of the control, sh-NC, and sh-LRP6 group after stretching for 24 h. Scale bar, 200 μm. *P < 0.05, **P < 0.01, ***P < 0.001. N.S., no significance. Results are presented as mean ± SD of triplicated experiments

LRP6 expression ascent in force-induced PDLSCs accompanied by the elevation of the proliferative and osteogenic capacity, manifested by the significantly increased expression of proliferation marker (PCNA) and osteogenic markers (ALP and RUNX2) (Fig. 2a, b). Next, we suppressed LRP6 expression via lentiviral vector construction to clarify the role of LRP6 on force-dependent osteogenic commitment in PDLSCs. The transfection efficiency was affirmed by fluorescence imaging (Additional file 2: Fig. S4). The protein levels of P-LRP6 and LRP6 were substantially downregulated in the sh-LRP6 group after stretching for 24 h (Fig. 2c). The same change in the mRNA expression was observed for *LRP6* (Fig. 2d), and an elevated ratio of P-LRP6/total LRP6 in the sh-LRP6 group indicated a slower decreasing trend of phosphorylation compared with total protein (Fig. 2e). Further, LRP6 knockdown attenuated the expression  of proliferation marker (PCNA) and osteogenic markers (ALP, RUNX2, OSX) (Fig. 2f, g). Concomitantly, a significantly declined ratio of Ki-67 positive cells (Fig. 2h, i) and dramatically weakened ALP staining (Fig. 2j) and ALP activity (Fig. 2k) in the sh-LRP6 group verified the proliferative and osteogenic ability reduction in LRP6-deficient cells. Our findings demonstrated that LRP6 was involved in modulating force-dependent proliferation and osteogenic differentiation of PDLSCs.

### LRP6 inactivation disrupted F-actin dynamics in force-induced PDLSCs

**Fig. 3:**
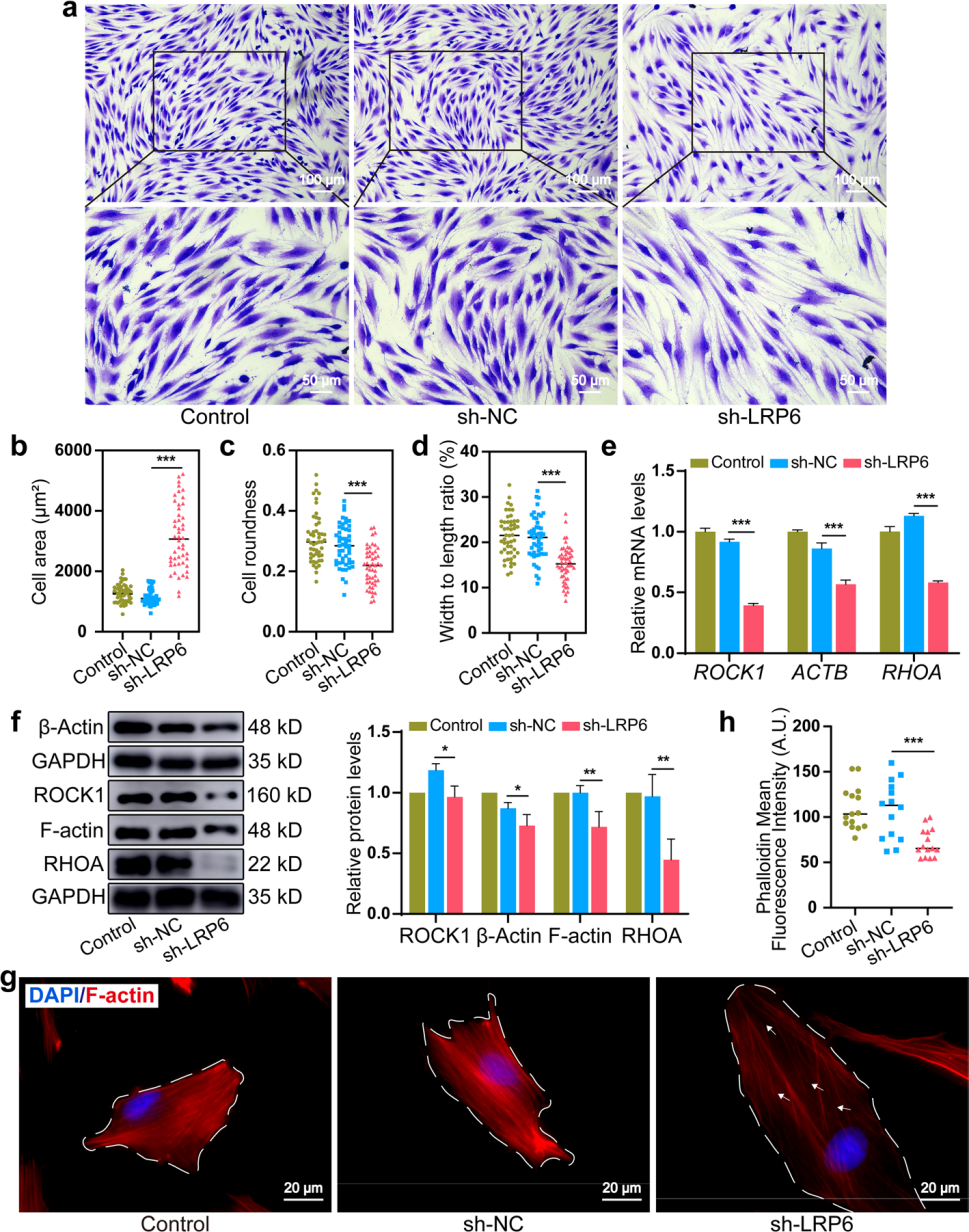
LRP6 inactivation disrupted F-actin dynamics in force-induced PDLSCs. **a** Imaging cell shape after stretching for 24 h by optical microscopy. Scale bars, 100 μm and 50 μm. **b** Quantification of cell area, **c** roundness, and **d** width-to-length ratio for the control, sh-NC, and sh-LRP6 group after stretching for 24 h. **e** The mRNA levels of *ROCK1*, *ACTB*, and *RHOA* in the control, sh-NC, and sh-LRP6 group after stretching for 24 h. **f** The protein levels of β-Actin, F-actin, ROCK1, and RHOA in the control, sh-NC, and sh-LRP6 group after stretching for 24 h. **g**, **h** Phalloidin fluorescence staining and semi-quantitative analysis in the control, sh-NC, and sh-LRP6 group after stretching for 24 h. Scale bar, 20 μm. *P < 0.05, **P < 0.01, ***P < 0.001. Results are presented as mean ± SD of triplicated experiments

Astonishingly, the aberrant changes in cell area and morphology were observed in the sh-LRP6 group, where LRP6-deficient cells displayed a significantly larger contact area, smaller cell roundness, and depressed width-to-length ratio after stretching for 24 h (Fig. 3a–d). Actin cytoskeleton rearrangement is necessary for the maintenance of force-induced cell shape [23, 24]. We thus assumed that LRP6 is a key regulator for F-actin dynamics in force-induced PDLSCs. Actually, β-Actin monomer synthesis and F-actin polymerization were severely suppressed (Fig. 3e, f) in the sh-LRP6 group under CSS. The expression of F-actin upstream regulators (RHOA and ROCK1), which is required for F-actin dynamics in force-induced PDLSCs [25], was also significantly decreased (Fig. 3e, f). Further, F-actin was disoriented and remarkably elongated (dotted lines in Fig. 3g), and the continuity of F-actin was apparently broken (arrows in Fig. 3g) in the sh-LRP6 group under CSS. Semi-quantification of phalloidin staining showed significant abatement of F-actin density in LRP6-deficient cells (Fig. 3h). These data indicated that LRP6 activation facilitates F-actin dynamics in force-induced PDLSCs and likely the structural homeostasis of PDL tissues during OTM.

### Disrupted F-actin dynamics suppressed osteogenic commitment in force-induced PDLSCs

**Fig. 4:**
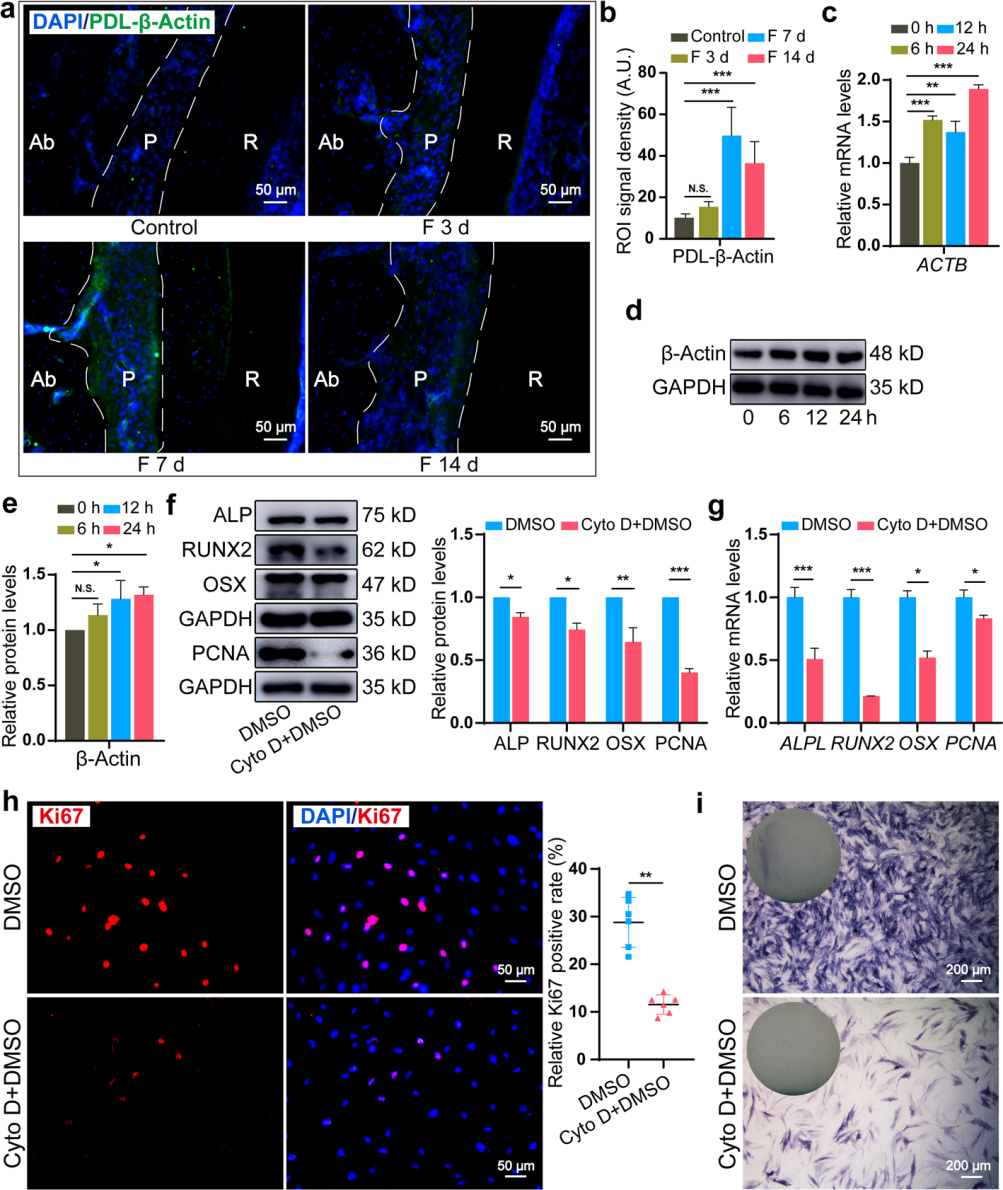
Disrupted F-actin dynamics suppressed osteogenic commitment in force-induced PDLSCs. **a**, **b** Immunofluorescence and semi-quantitative analysis of β-Actin expression in the periodontium during mechanical force application. *N* = 5. Ab, alveolar bone; P, periodontal tissue; R, root. Dotted lines mark the boundary of the PDL. Scale bar, 50 μm. **c** The mRNA expression of *ACTB* in PDLSCs during CSS loading. **d**, **e** The protein expression of β-Actin in PDLSCs during CSS loading. **f** The protein expression of ALP, RUNX2, OSX, and PCNA in the DMSO and Cyto D + DMSO groups after stretching for 24 h. **g** The mRNA expression of *ALPL*, *RUNX2*, *OSX*, and *PCNA* in the DMSO and Cyto D + DMSO groups after stretching for 24 h. **h** Immunofluorescence and semi-quantitative analysis of Ki67^+^ cells in the DMSO and Cyto D + DMSO groups after stretching for 24 h. Scale bar, 50 μm. **i** ALP staining in the DMSO and Cyto D + DMSO groups after stretching for 24 h. Scale bar, 200 μm. *P < 0.05, **P < 0.01, ***P < 0.001. N.S., no significance. Results are presented as mean ± SD of triplicated experiments

Then we examined whether LRP6-mediated F-actin dynamics participate in force-dependent osteogenic commitment. We first detected β-Actin expression in force-induced PDLSCs and PDL tissues. The expression of β-Actin, which is consistent with the LRP6 expression pattern, showed significant accumulation from day 0 to day 7 and a slight retraction on day 14 in vivo (Fig. 4a, b) and steadily raised after being stretched in vitro (Fig. 4c–e). The data further verified that LRP6 deficiency in PDLSCs disrupted force-induced F-actin assembly. Next, we studied the role of F-actin dynamics on force-dependent osteogenic commitment in PDLSCs using Cyto D, a potent actin polymerization inhibitor. As shown in Fig. 4f, g, Cyto D prevented the expression of ALP, RUNX2, OSX, and PCNA, accompanied by β-Actin expression inhibition (Additional file 2: Fig. S5). Then, a strikingly decreased ratio of Ki-67-positive cells (Fig. 4h) and weakened ALP staining (Fig. 4i) caused by Cyto D verified the attenuated ability of force-dependent osteogenic commitment in PDLSCs due to F-actin dynamics disruption. Accordingly, we determined that F-actin acts as a key downstream branch of the LRP6 signaling regulate force-dependent osteogenic commitment in PDLSCs.

### LRP6/F-actin axis modulated YAP nuclear translocation and activation in force-induced PDLSCs

**Fig. 5:**
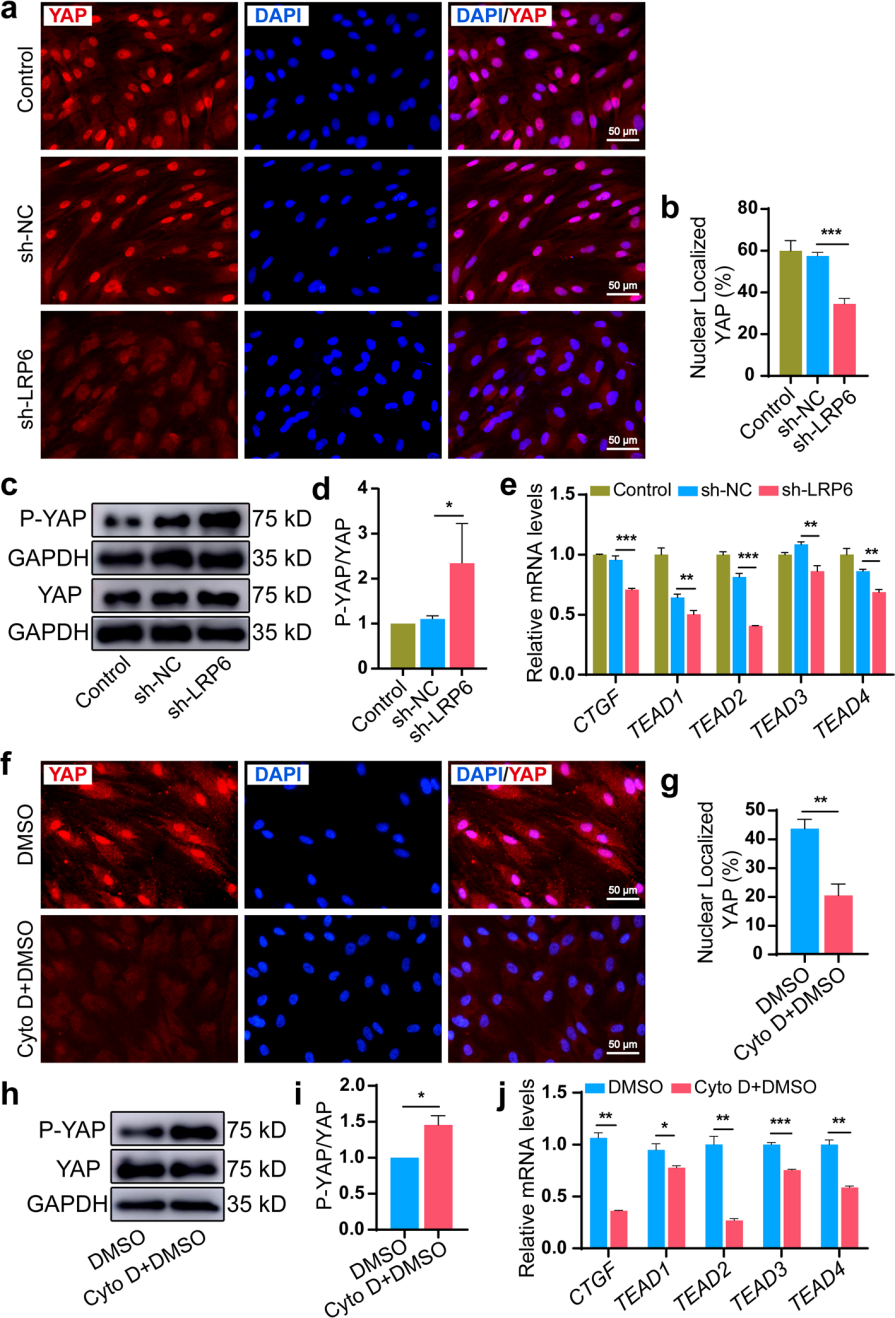
LRP6/F-actin axis modulated YAP nuclear translocation and activation in force-induced PDLSCs. **a**, **b** Immunofluorescence and semi-quantitative analysis of YAP nuclear localization in the control, sh-NC, and sh-LRP6 group after stretching for 24 h. Scale bar, 50 μm. **c**, **d** Western blotting analysis indicated the ratio of P-YAP/total-YAP in the control, sh-NC, and sh-LRP6 group after stretching for 24 h. **e** The mRNA levels of YAP target genes (*CTGF*, *TEAD1*, *TEAD2*, *TEAD3*, *TEAD4*) in the control, sh-NC, and sh-LRP6 group after stretching for 24 h. **f**, **g** Immunofluorescence and semi-quantitative analysis of YAP nuclear localization in the DMSO and Cyto D + DMSO groups after stretching for 24 h. Scale bar, 50 μm. **h**, **i** Western blotting analysis indicated the ratio of P-YAP/total-YAP in the DMSO and Cyto D + DMSO groups after stretching for 24 h. **j** The mRNA levels of YAP target genes (*CTGF*, *TEAD1*, *TEAD2*, *TEAD3*, *TEAD4*) in the DMSO and Cyto D + DMSO groups after stretching for 24 h. *P < 0.05, **P < 0.01, ***P < 0.001. Results are presented as mean ± SD of triplicated experiments

**Fig. 6:**
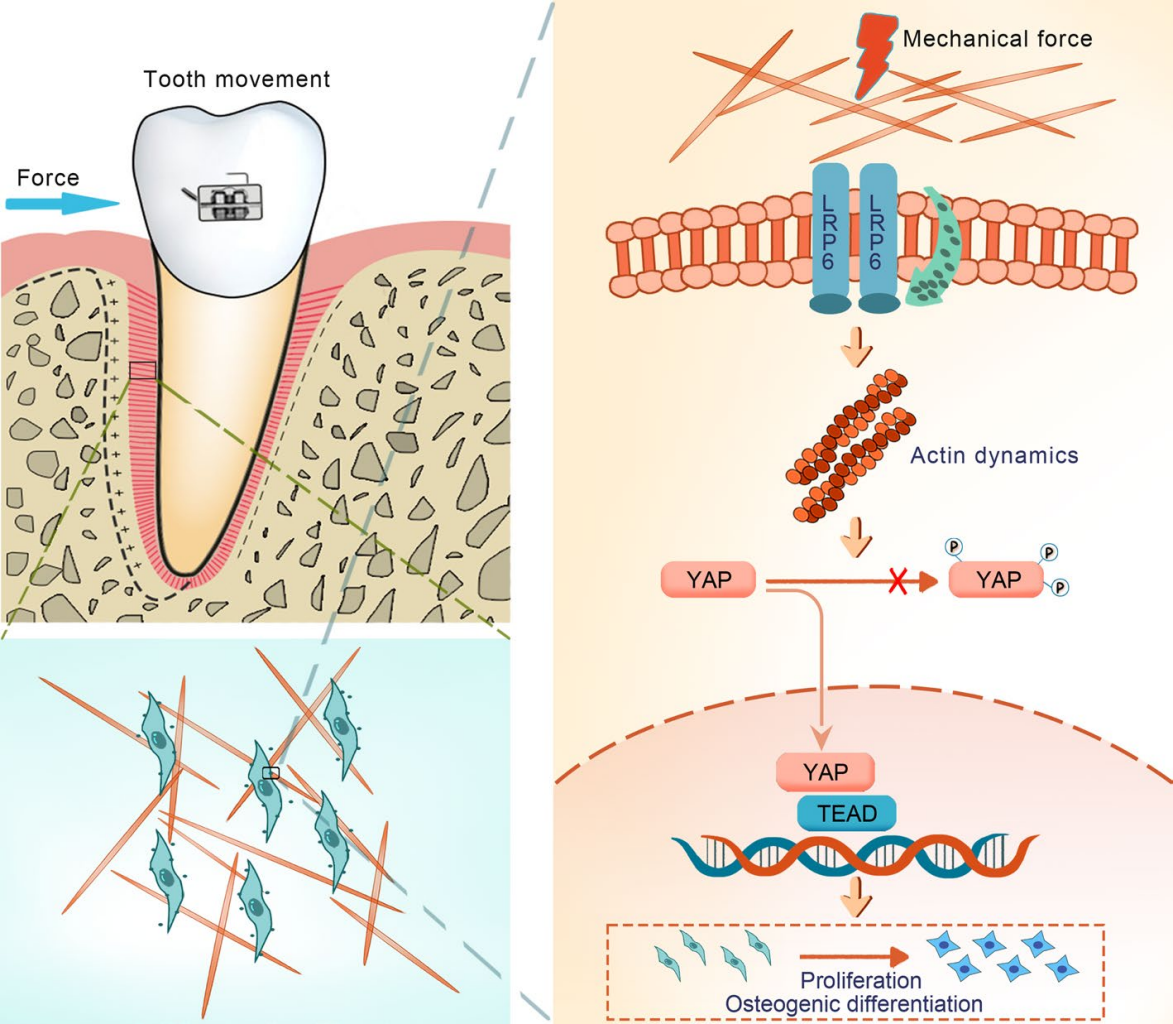
Schematic diagram for the proposed mechanism. During alveolar bone remodeling that is initiated by orthodontic application, mechanical force enhances LRP6 membrane localization in PDLSCs. LRP6 mobilization then facilitates F-actin polymerization and rearrangement and further boosts YAP shuttling to the nucleus. Activated YAP subsequently promotes cell proliferation and osteogenic differentiation and ultimately leads to new bone formation

YAP acts by shuttling into the nucleus and is a critical transcriptional mediator for force-dependent osteogenic commitment in PDLSCs [26, 27]. We speculated that YAP acts as a key transducer coordinated with the signal from the LRP6/F-actin axis and finally contributes to force-dependent osteogenic commitment in PDLSCs. We first examined the relation between LRP6 expression and YAP localization, phosphorylation, and transcriptional activity. LRP6 knockdown relocated nuclear YAP to the cytoplasm, although YAP was mainly concentrated in the nucleus after CSS loading (Fig. 5a, b). Meanwhile, we observed that LRP6 knockdown induced YAP phosphorylation (Fig. 5c, d). Significantly, the expression of YAP target genes was dramatically diminished in the LRP6-deficient cells (Fig. 5e). Moreover, Cyto D also relocated nuclear YAP to the cytoplasm in force-induced PDLSCs (Fig. 5f, g), and its usage induced YAP phosphorylation (Fig. 5h, i) and decreased YAP target gene expression (Fig. 5j). The results implied that LRP6 knockdown in PDLSCs disturbed F-actin dynamics, further regulating YAP nuclear translocation and subsequent transcriptional response (Fig. 6).

## Discussion

The prerequisite for alveolar bone remodeling is the osteogenesis regulated by mechanotransduction of mechanically induced PDLSCs during OTM [28]. The transmission of mechanical information concerns mechanosensing and ensuing signaling [29]. During the past years, the contribution of mechanosensors as mechanosensing hubs to mechanotransduction events was increasingly valued [30]. An improved understanding of sensor-regulated mechanotransduction of PDLSCs holds great promise for engineering mechanotargeting interventions to optimize osteogenic activities. Therefore, this work focused on the proximal events detailing the process from mechanical inputs to transcriptional outputs.

LRP6, a type I transmembrane protein that ubiquitously expresses in human tissues, has been verified to be vital for tissue development and skeletal homeostasis [31, 32]. For instance, LRP6 dysregulation can cause defective osteoblasts function but high bone mass due to LRP6 mutation [33, 34]. Recently, more attention has been attracted to studying the relationship between LRP6 and tooth/craniofacial development owing to several case reports about tooth agenesis and cleft lip caused by mutations in the *LRP6* gene [35, 36]. Excitingly, the critical role of LRP6 for epithelial–mesenchymal interactions during molar morphogenesis and the endothelial differentiation of dental pulp stem cells has been elucidated [17, 37]. These findings prompt the potential significance of LRP6 in alveolar bone remodeling during OTM. Since alveolar bone remodeling occurs in the initial stage of OTM [26], the applied force timeframe was designed to reflect the initial phase. We first verified that CSS (10% elongation, 0.5 Hz) is preferable to boost osteoblastic differentiation and proliferation, which accords with the peculiarity of the initial phase of OTM [38]. Then, we confirmed a significant positive correlation between the LRP6 amount and applied-force time in vitro. The slight decrease at 6 h may be attributed to retardant protein expression. In vivo, we observed that LRP6 was widespread in the periodontium and the expression pattern of LRP6 corresponds with the feature of OTM, which consists of an early responsive stage and a subsequent adaptive stage. Together, the augmented expression indicated the increased demands for LRP6 in force-induced PDLSCs during OTM. Moreover, LRP6 inhibition distinctly impaired osteogenic differentiation, manifested by weaker ALP activity and lower expression levels of osteogenic markers in the sh-LRP6 group under CSS. Similarly, LRP6 deficiency on the membrane of bone marrow mesenchymal stem cells(MSCs) restrains the development into the osteoblastic lineage, contributing to atherosclerosis-related bone loss [39]. Noteworthily, the high ALP activity was detected in the control and sh-NC groups as early as 24 h after stretching, which is significantly sooner than in PDLSCs treated with osteogenic medium. The early and rapid response of ALP to mechanical stress was also reported by other teams [40, 41]. This could indicate that the effect of mechanical force as the stimulus appears to accelerate the osteogenic differentiation of PDLSCs to osteoblasts. Simultaneously, the negative connection between LRP6 loss and proliferation demonstrates that LRP6 is necessary for force-dependent proliferation, which parallels the pivotal role of LRP6 for hematopoietic and intestinal stem cells [42, 43]. Overall, LRP6 activation is responsible for osteogenic activities in mechanically induced PDLSCs. Besides, the incremental expression of LRP5 under CSS implied that LRP5, as an LRP6 ortholog, could perform a compensatory role as observed during mouse tooth development and intestinal development [17, 44]. These results endorsed LRP6 as a CSS mechanosensor.

Notably, we clarified that LRP6 functions as a regulator for F-actin dynamics under CSS. We were astonished by the aberrant alteration of cell morphology in LRP6-deficient cells after CSS loading. Moreover, cell morphogenetic alterations are mediated by mechanically induced actin cytoskeleton dynamics. Indeed, we observed β-Actin synthesis reduction and F-actin depolymerization with remarkable disorientation after LRP6 knockdown, although β-Actin polymerization was pronouncedly reinforced during CSS loading. It should be noted that the regulation of LRP6 on F-actin dynamics was observed only during neural tube closure and colorectal cancer progression [45, 46]. Besides, the fact that actin-dependent LRP6 endocytosis is a key procedure for neuroepithelial proliferation [47] reminds us that there may be a situational regulation between LRP6 and actin cytoskeleton. Further, the propagation of mechanosensing information in regulating cell behavior (e.g., proliferation, differentiation, migration) engages mechanically actuated F-actin dynamics [48, 49]. In fact, LRP6 loss hindered F-actin dynamics, and subsequent disruption of F-actin dynamics crippled elevated proliferative and osteogenic capacity in CSS-induced PDLSCs. This suggested tthat F-actin acts as a crucial mechanotransducer for LRP6-regulated mechano-cascading. These findings lend new mechanistic insight into the functional dynamics of actin cytoskeleton ruling mechano-driven MSCs fate decisions.

YAP, a vital nuclear effector of the Hippo pathway, is under spatiotemporal regulation of its mechanical strains linked to the local inputs, which are dictated by F-actin dynamics [50]. YAP stimulation has been associated with force-dependent osteogenic commitment in PDLSCs [26, 27]. Moreover, we verified that turning on YAP is controlled by the F-actin dynamics, which is in turn decided by LRP6 expression under CSS. Indeed, many membrane receptors, such as integrin and G-protein-coupled receptors, are key regulators for YAP activation through F-actin remodeling [51, 52]. Different membrane receptors that are likely coupled to corresponding ligands synergistically regulate YAP activity and further fine-tune the osteogenic program in mechanically induced PDLSCs. However, few upstream control switches presiding over YAP signaling have been proposed so far. The findings provide a complement for YAP regulatory pathways devoted to alveolar bone remodeling control. Previous studies have reported that LRP6 either directly regulates YAP activity by Hippo signaling or acts as a checkpoint coordinate YAP/WNT signaling [53–55]. Our findings advance the understanding of LRP6-YAP signaling interplay. Nevertheless, whether YAP activation/inhibition is a potent approach to treat skeletal diseases by dysregulated LRP6 needs further investigation. Future work will focus on mapping the detailed mechanism of the LRP6/F-actin signaling cascade. On the other hand, the Wnt/β-Catenin pathway, the primary downstream branch of LRP6 signaling, is a key determinant of PDLSCs mechanotransduction [56]. LRP6 phosphorylation initiates the downstream β-Catenin pathway [57]. Indeed, force application elevated LRP6 phosphorylation and the expression of β-Catenin was inactivated in LRP6-deficient PDLSCs under CSS (Additional file 2: Fig. S6). This indicated the potential role of LRP6 phosphorylation in LRP6-regulated signaling. Although LRP6 deficiency inhibited β-Catenin activation under CSS, we here emphasized a unique route for LRP6 outside the canonical pathway to mediate the mechanotransduction of PDLSCs .

## Conclusions

This study revealed a distinct mechanotransduction machinery for controlling osteogenic commitment, centered on LRP6, connecting mechanical cues to the F-actin/YAP cascade in mechanically induced PDLSCs. These results shed light on the molecular basis of OTM.

## Supplementary Information

**Additional file 1: Table S1.** Primer sequences for qRT-PCR. **Table S2.** Primary antibodies for western blotting and immunofluorescence.

**Additional file 2: Fig. S1.** The intraoral picture of the experimental OTM model. **Fig. S2.** Representative images of HE staining of the PDL on the tension side of M1 mesiobuccal roots. Ab, alveolar bone; P, periodontal tissue; R, root. Scale bar, 50 μm. **Fig. S3.** The protein expression of LRP5 in PDLSCs during CSS loading. **Fig. S4.** Fluorescence imaging showing the efficiency of lentiviral transfection. **Fig. S5.** The protein expression of β-Actin in the DMSO and Cyto D + DMSO groups after stretching for 24 h. **Fig. S6.** LRP6 inactivation suppressed β-Catenin expression in force-induced PDLSCs. **a** The protein expression of β-Catenin and active β-Catenin in the control, sh-NC, and sh-LRP6 group after stretching for 24 h. **b** The mRNA expression of *β-Catenin* in the control, sh-NC, and sh-LRP6 group after stretching for 24 h. *P < 0.05, ***P < 0.001. N.S., no significance. Results are presented as mean ± SD of triplicated experiments. **Fig. S7** Culture and identification of PDLSCs. **a** Cell shape of the first (P0) and the third passage (P3) of PDLSCs. Scale bar, 200 μm. **b** Monoclonal formation after cultured cells were incubated for 14 days. Scale bar, 100 μm. **c** Expression of mesenchymal stem cell markers (CD90, STRO-1, and CD146) and leukocytic and hematopoietic cell markers (CD45 and CD34) detected by flow cytometry. **d** Alizarin Red staining of cultured cells in the control group and osteogenic induction group. Scale bar, 100 μm. **e** Oil Red O staining of cultured cells in the control group and adipogenic induction group. Scale bar, 50 μm.

**Additional file 3. **Original images of western blotting.

## Data Availability

The datasets used and/or analyzed during the current study are available from the corresponding author on reasonable request.

## Abbreviations

PDLSCs: Periodontal ligament stem cells
PDL: Periodontal ligament
LRP6: Low-density lipoprotein receptor-related protein 6
LRP5: Low-density lipoprotein receptor-related protein 5
F-actin: Filamentous actin
OTM: Orthodontic tooth movement
CSS: Cyclic stretch stress
Cyto D: Cytochalasin D
qRT-PCR: Quantitative real-time polymerase chain reaction
ALP: Alkaline phosphatase
HE: Hematoxylin–eosin
MSCs: Mesenchymal stem cells
