# Supplementary material for: LRP6/filamentous-actin signaling facilitates osteogenic commitment in mechanically induced periodontal ligament stem cells
Source: Cell Mol Biol Lett. 2023 Jan 24;28:7. doi: 10.1186/s11658-023-00420-5 (PMC9872397; doi:10.1186/s11658-023-00420-5)
Supplement: Supplementary file 1 — Additional file 1: Table S1. Primer sequences for qRT-PCR. Table S2. Primary antibodies for western blotting and immunofluorescence. [file 11658_2023_420_MOESM1_ESM.pdf]

Additional file 1

Table S1. Primer sequences for qRT-PCR

Name	Forward (5'-3')	Reverse (5'-3')
LRP6	AGACGGGACTTGCGATTGG	TCCTCCAAGCCTCCAACTACA
ROCK1	GGAAGTGAGGTTAGGGCGAA	ACAGTGTCTCGGAGCGTTTC
CTNNB1	GCTGCAACTAAACAGGAAGGG	CCCACTTGGCAGACCATCAT
ALPL	CCACGTCTTCACATTTGGTG	AGACTGCGCCTGGTAGTTGT
RUNX2	CGAATTGGCAGCACGCTATTAA	GTCGCCAAACAGATTCATCCA
OSX	ATCCAGCCCCCTTTACAAGC	TAGCATAGCCTGAGGTGGGT
ACTB	CATGTACGTTGCTATCCAGGC	CTCCTTAATGTCACGCACGAT
PCNA	TCTGAGGGCTTCGACACCTA	TCTCCTGGTTTGGTGCTTCA
GAPDH	GCACCGTCAAGGCTGAGAAC	TGGTGAAGACGCCAGTGGA
RHOA	AGCCGGTGAAACCTGAAGAA	GCTGAACACTCCATGTACCCA
CTGF	TTAGCGTGCTCACTGACCTG	GCCACAAGCTGTCCAGTCTA
TEAD1	CTCAGGACAGGCAAGACGAG	TTATGAATGGCAGTGGCCGA
TEAD2	ATGAACTGATCGCCCGCTAC	TTGCCATTGTCTGGAAAGCC
TEAD3	CAGGACCGTACCATTGCCTC	GCTGTACGTGTCAGGGTCTC
TEAD4	CTCCAGCCAGTATGAGAGCC	ATGTACTCACAGAGCGGGGA

Table S2. Primary antibodies for Western blotting and Immunofluorescence

Name	
LRP6	Cell Signaling Technology, #2560
LRP6	Santa Cruz Biotechnology, sc-25317
Phospho-LRP6	Cell Signaling Technology, #2568
LRP5	Abcam, ab223203
ROCK1	Rho-associated protein kinase 1, Abcam, ab134181
Ki-67	Proliferation maker protein Ki-67, Cell Signaling Technology, #9449
YAP	Yes-associated protein, Cell Signaling Technology, #14074
Phospho-YAP	Abcam, ab76252
ALP	Alkaline phosphatase, biomineralization associated, HUABIO, SA40-00
active β-Catenin	Abcam, ab246504
β-Catenin	Abcam, ab32572
RUNX2	Runt-related transcription factor 2, Cell Signaling Technology, #8486
OSX	Osterix, Abcam, ab209484
F-actin	Abcam, ab130935
β-Actin	Proteintech, 20536-1-AP
PCNA	Proliferating cell nuclear antigen, Cell Signaling Technology, #2586
GAPDH	Glyceraldehyde 3-phosphate dehydrogenase, Abcam, ab181602
RHOA	Ras homolog gene family, member A, Abcam, ab187027
