# Supplementary material for: LRP6/filamentous-actin signaling facilitates osteogenic commitment in mechanically induced periodontal ligament stem cells
Source: Cell Mol Biol Lett. 2023 Jan 24;28:7. doi: 10.1186/s11658-023-00420-5 (PMC9872397; doi:10.1186/s11658-023-00420-5)
Supplement: Supplementary file 2 — Additional file 2: Fig. S1. The intraoral picture of the experimental OTM model. Fig. S2. Representative images of HE staining of the PDL on the tension side of M1 mesiobuccal roots. Ab, alveolar bone; P, periodontal tissue; R, root. Scale bar, 50 μm. Fig. S3. The protein expression of LRP5 in PDLSCs during CSS loading. Fig. S4. Fluorescence imaging showing the efficiency of lentiviral transfection. Fig. S5. The protein expression of β-Actin in the DMSO and Cyto D + DMSO groups after stretching for 24 h. Fig. S6. LRP6 inactivation suppressed β-Catenin expression in force-induced PDLSCs. a The protein expression of β-Catenin and active β-Catenin in the control, sh-NC, and sh-LRP6 group after stretching for 24 h. b The mRNA expression of β-Catenin in the control, sh-NC, and sh-LRP6 group after stretching for 24 h. *P < 0.05, ***P < 0.001. N.S., no significance. Results are presented as mean ± SD of triplicated experiments. Fig. S7 Culture and identification of PDLSCs. a Cell shape of the first (P0) and the third passage (P3) of PDLSCs. Scale bar, 200 μm. b Monoclonal formation after cultured cells were incubated for 14 days. Scale bar, 100 μm. c Expression of mesenchymal stem cell markers (CD90, STRO-1, and CD146) and leukocytic and hematopoietic cell markers (CD45 and CD34) detected by flow cytometry. d Alizarin Red staining of cultured cells in the control group and osteogenic induction group. Scale bar, 100 μm. e Oil Red O staining of cultured cells in the control group and adipogenic induction group. Scale bar, 50 μm. [file 11658_2023_420_MOESM2_ESM.docx]

**Additional file 2**


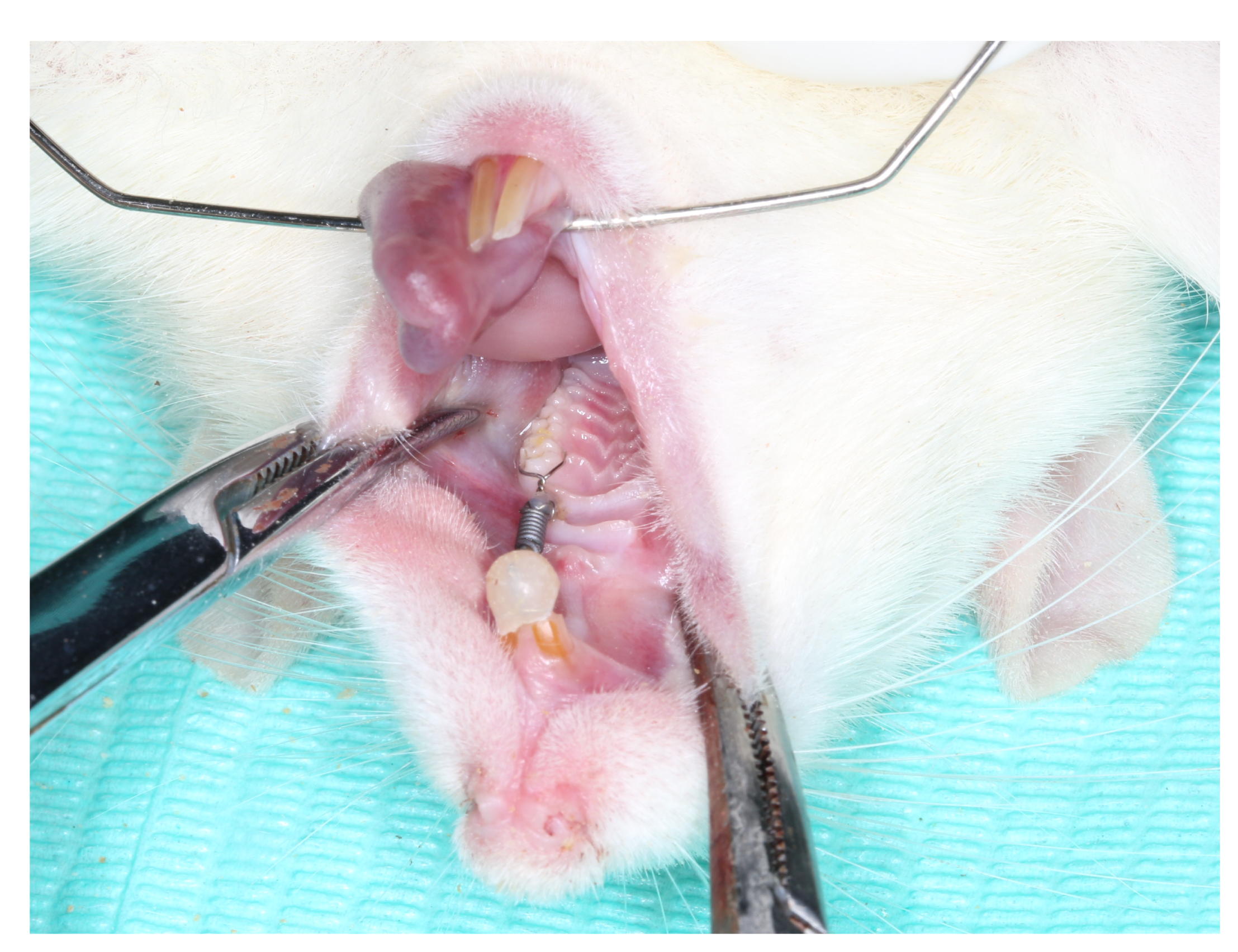


**Fig. S1** The intraoral picture of the experimental OTM model.


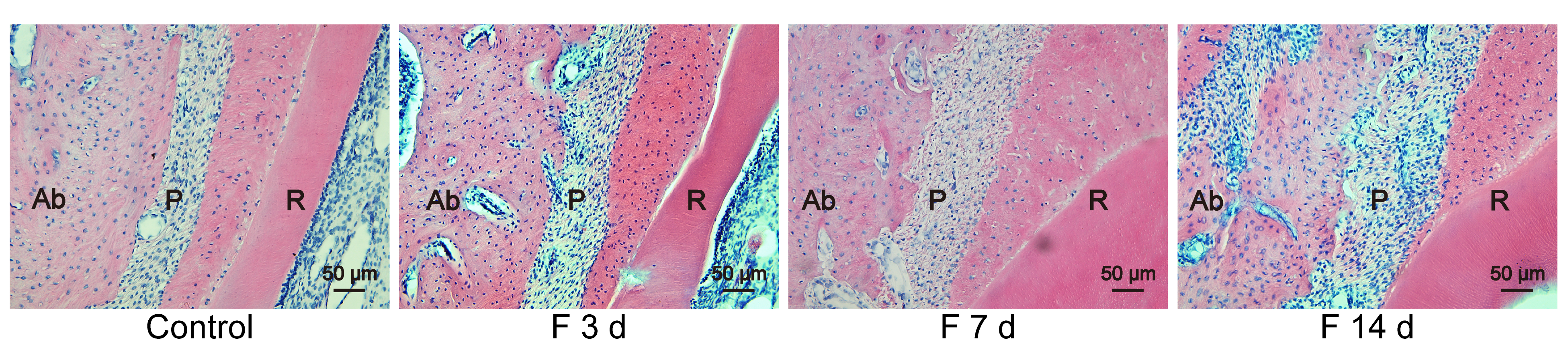


**Fig. S2** Representative images of HE staining of the PDL on the tension side of M1 mesiobuccal roots.


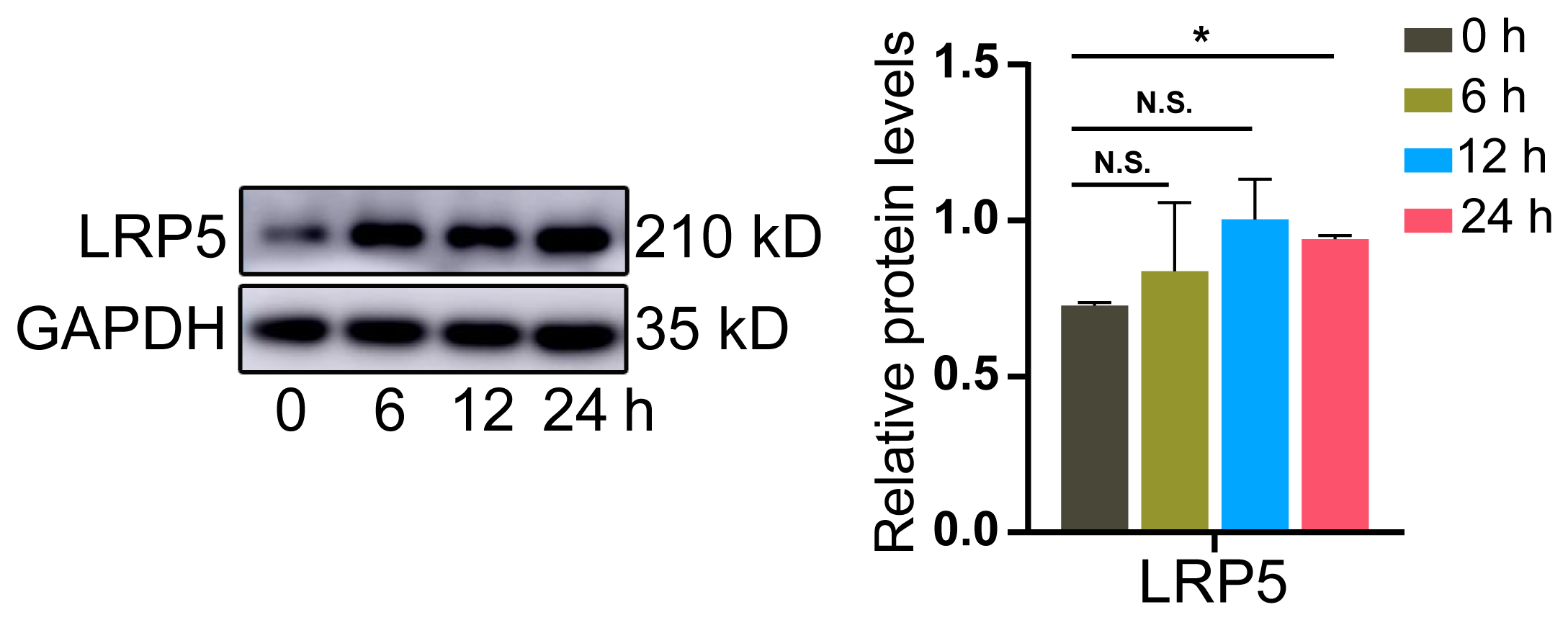


**Fig. S3** The protein expression of LRP5 in PDLSCs during CSS loading.


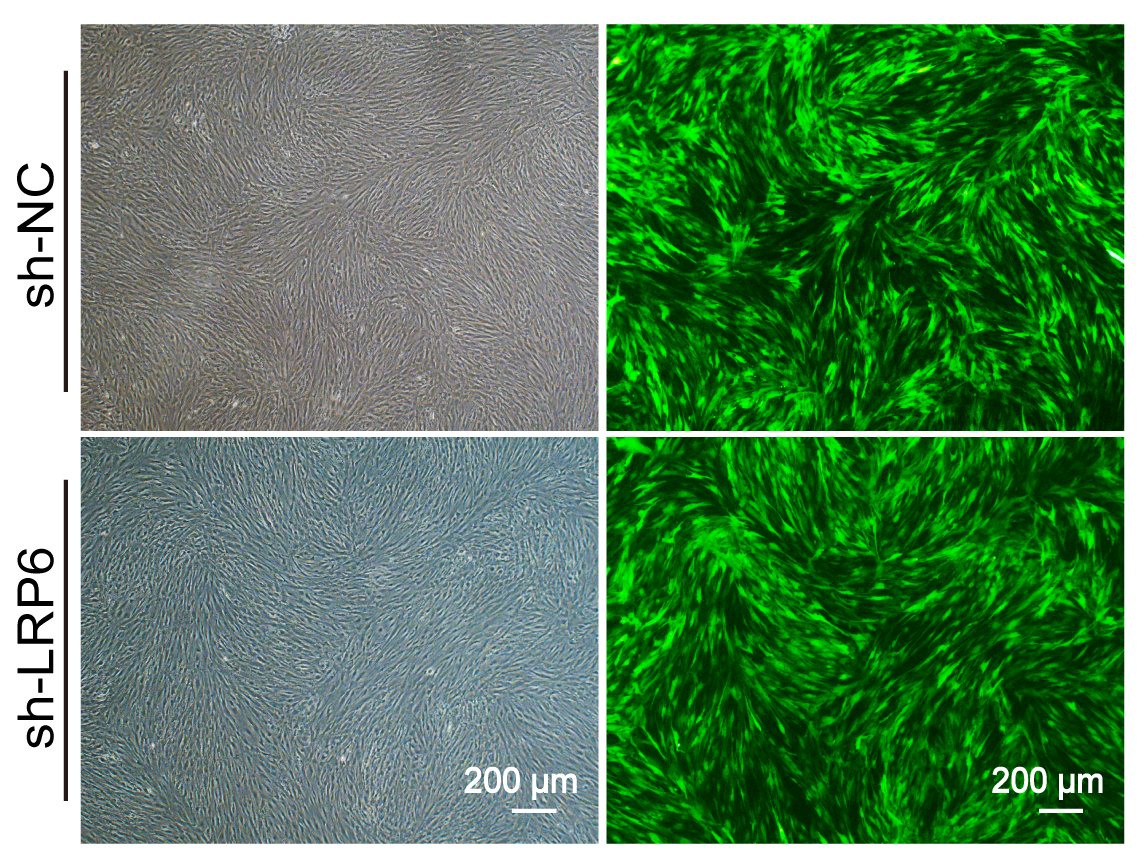


**Fig. S4** Fluorescence imaging showed the efficiency of lentiviral transfection.

**
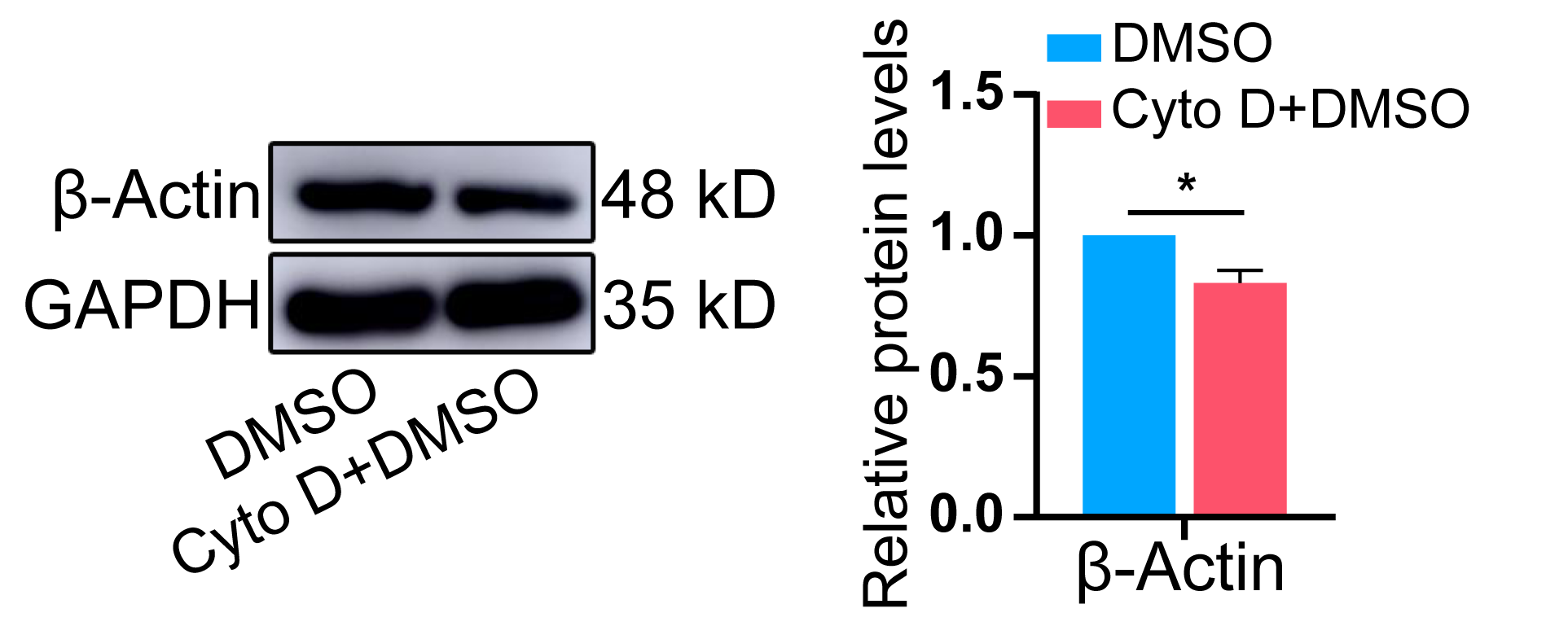
**

**Fig. S5** The protein expression of β-Actin in the DMSO and Cyto D+DMSO group after stretching for 24 h.


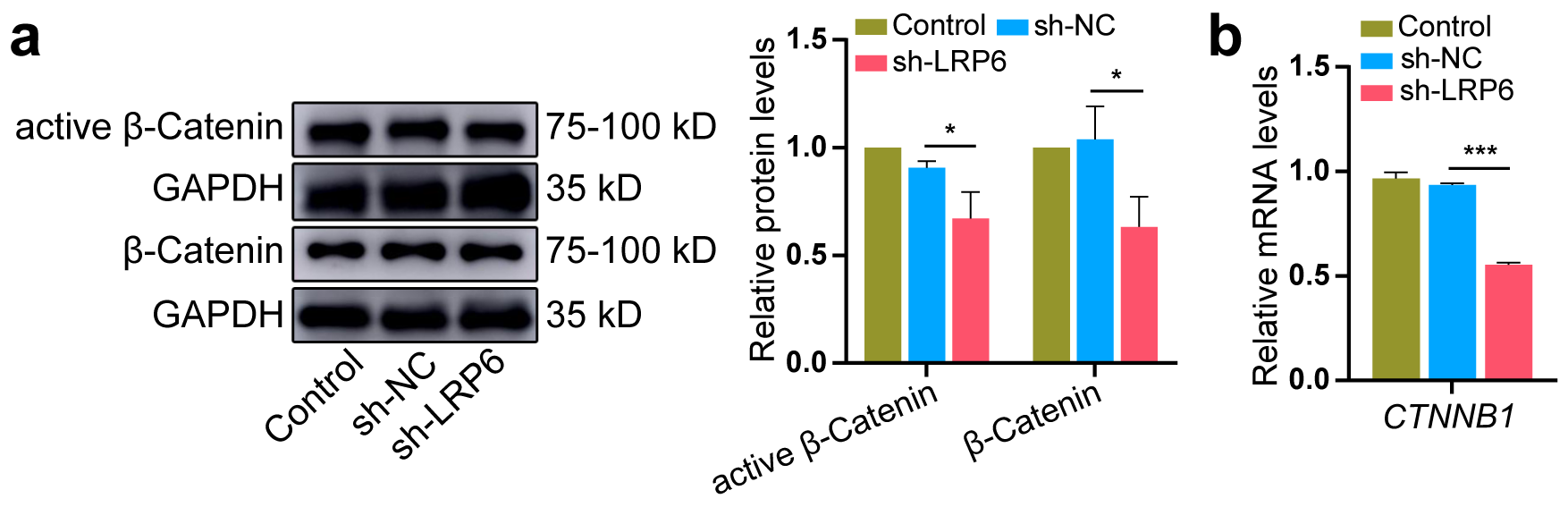


**Fig. S6** LRP6 inactivation suppressed β-Catenin expression in force-induced PDLSCs.


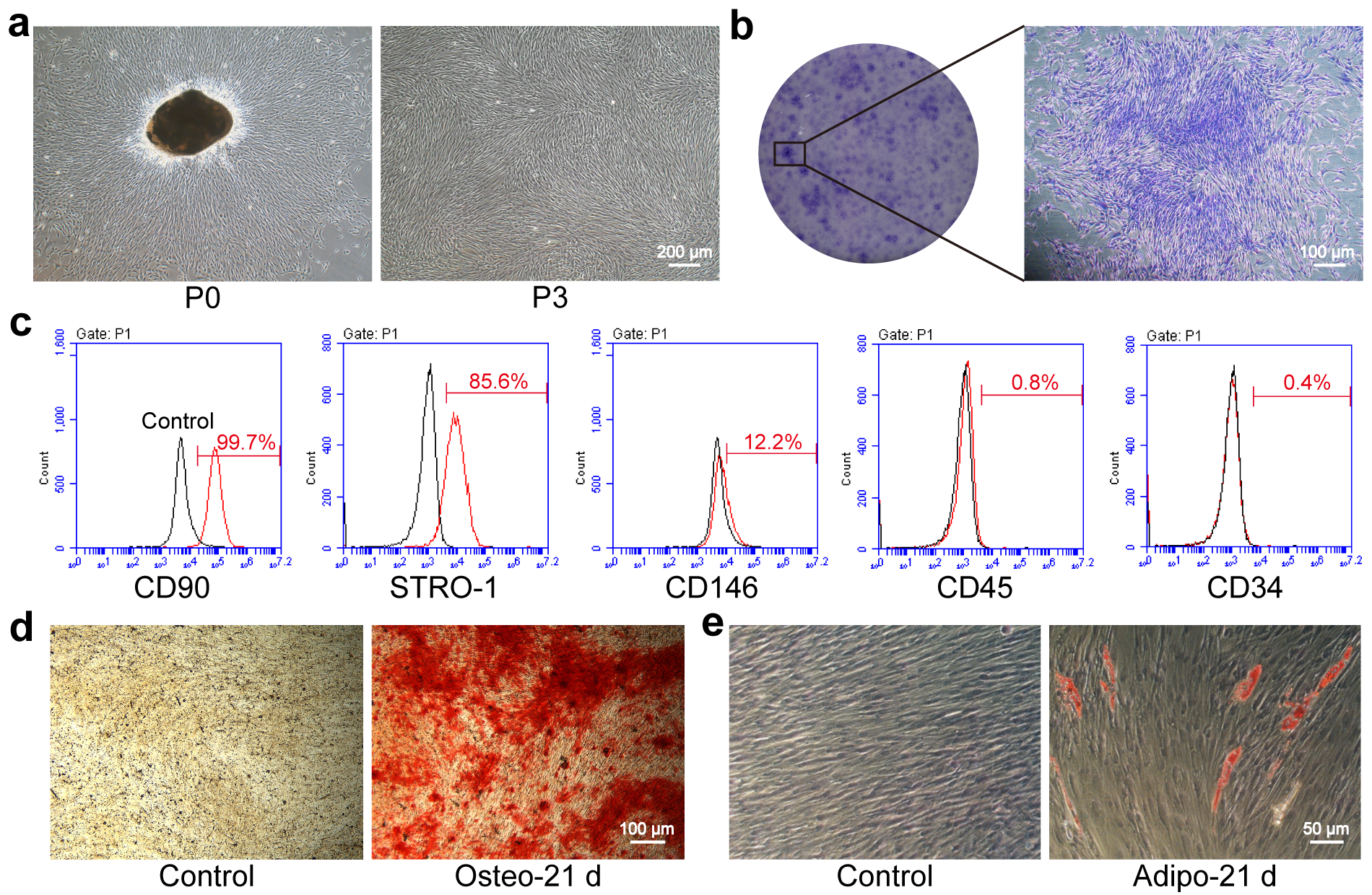


**Fig. S7** Culture and identification of PDLSCs.
