# Supplementary figures and images for: LRP6/filamentous-actin signaling facilitates osteogenic commitment in mechanically induced periodontal ligament stem cells
Source: Cell Mol Biol Lett. 2023 Jan 24;28:7. doi: 10.1186/s11658-023-00420-5 (PMC9872397; doi:10.1186/s11658-023-00420-5)

**Additional file 3**

**
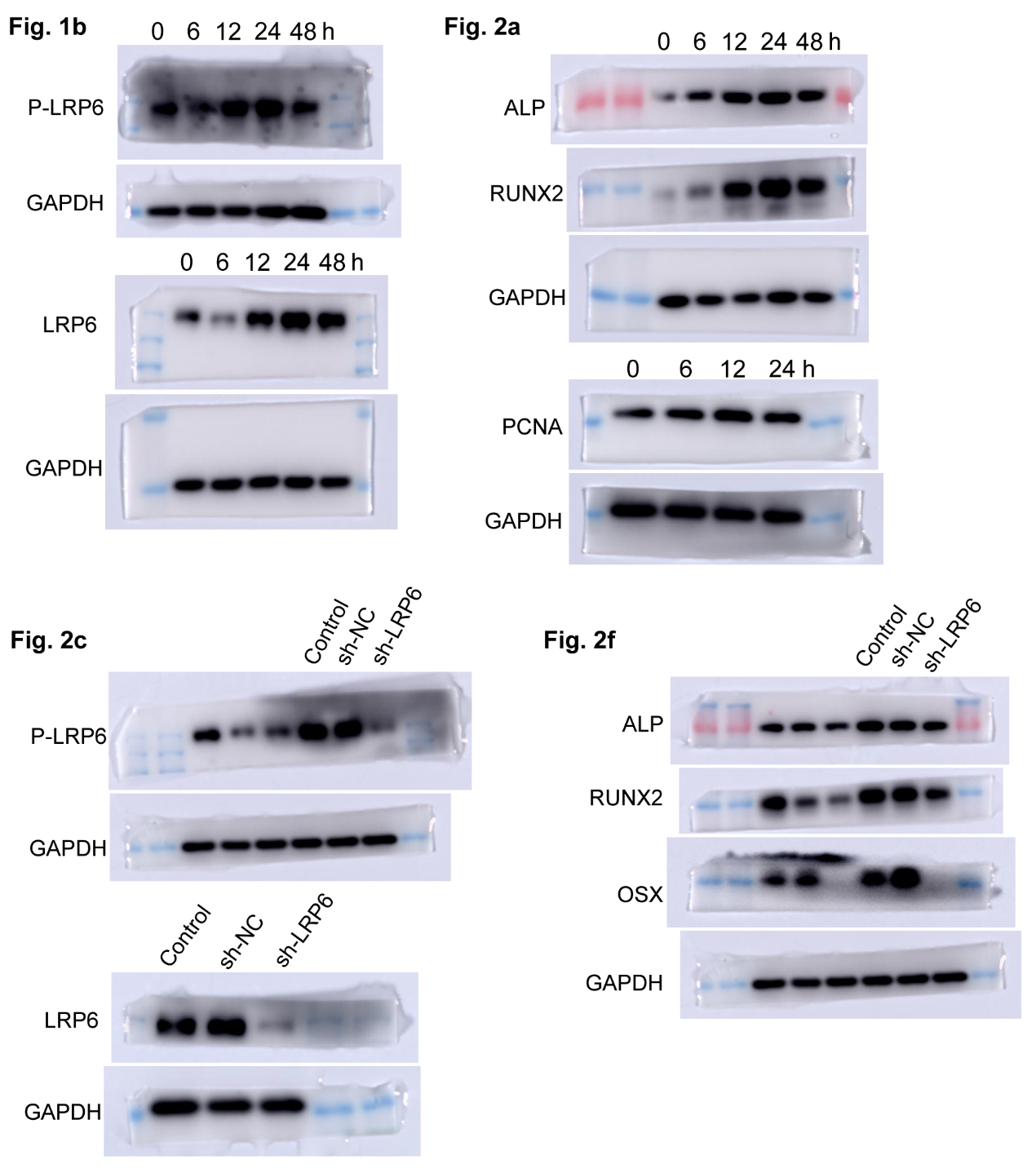
**

**
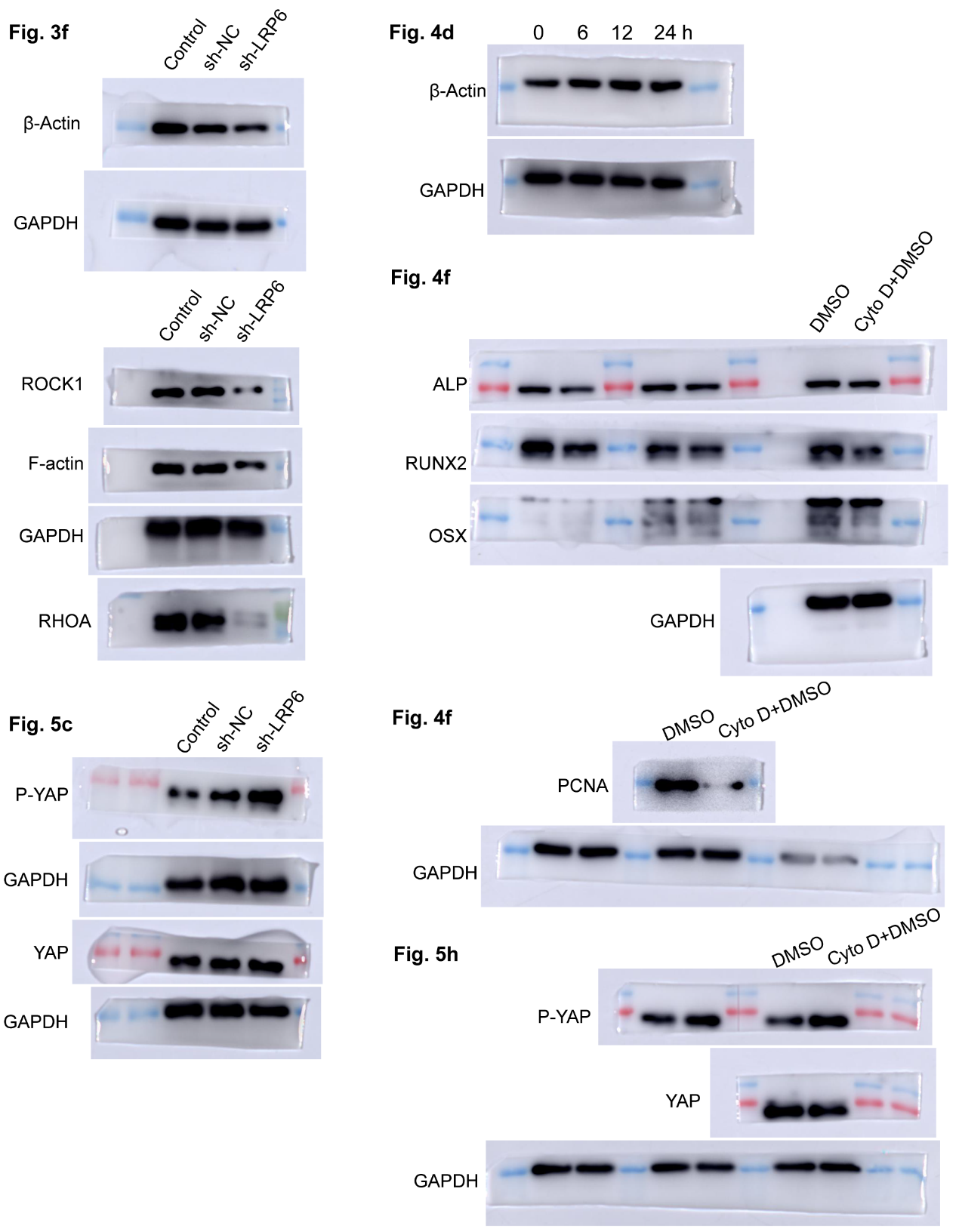
**

**
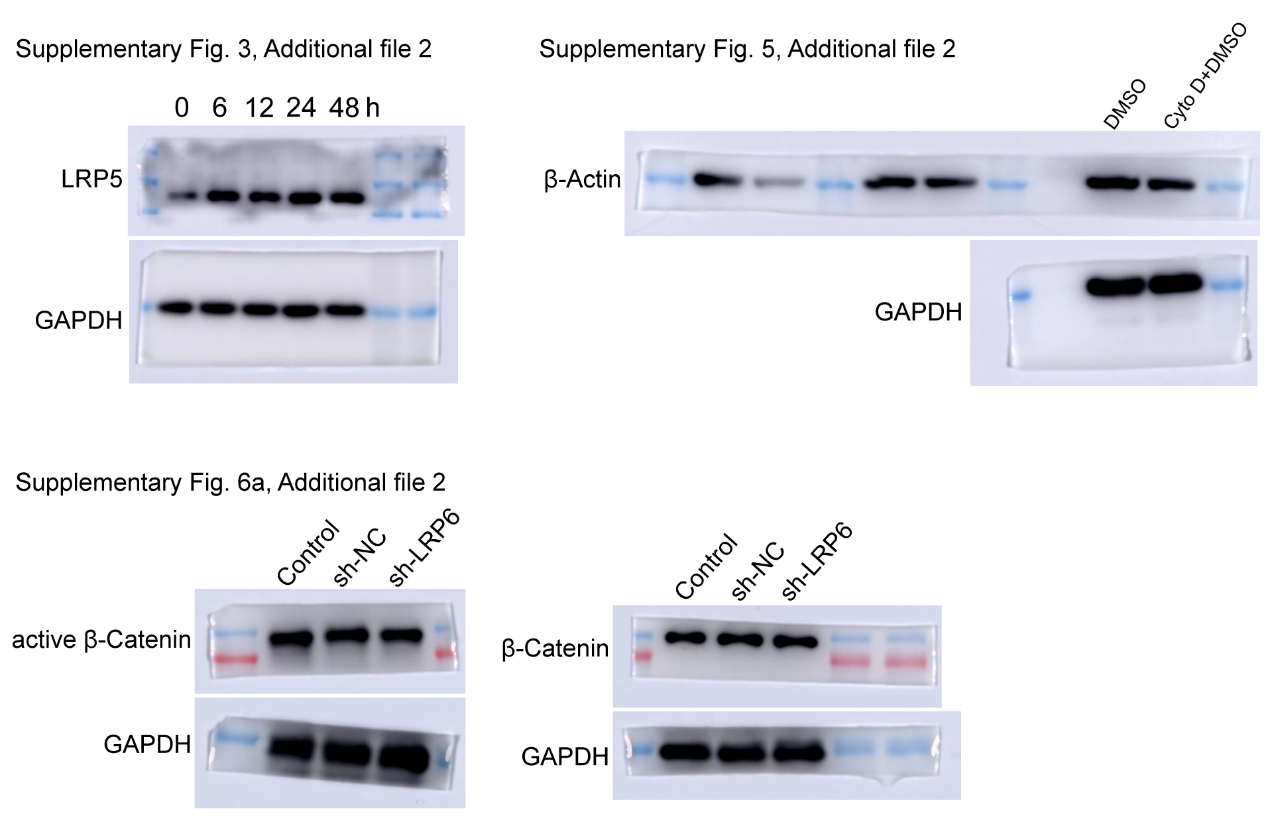
**

Supplement: Supplementary file 3 — Additional file 3. Original images of western blotting. [file 11658_2023_420_MOESM3_ESM.docx]
